# A World-First Surgical Instrument for Minimally Invasive Robotically-Enabled Transplantation of Heart Patches for Myocardial Regeneration: A Brief Research Report

**DOI:** 10.3389/fsurg.2021.653328

**Published:** 2021-10-06

**Authors:** Christopher David Roche, Yiran Zhou, Liang Zhao, Carmine Gentile

**Affiliations:** ^1^Northern Clinical School of Medicine, University of Sydney, Sydney, NSW, Australia; ^2^School of Biomedical Engineering, Faculty of Engineering and IT, University of Technology Sydney, Sydney, NSW, Australia; ^3^Department of Cardiothoracic Surgery, University Hospital of Wales, Cardiff, United Kingdom; ^4^School of Mechanical and Mechatronic Engineering, Faculty of Engineering and IT, University of Technology Sydney, Sydney, NSW, Australia

**Keywords:** instrumentation, regeneration, thorascopic surgery, myocardial patch, automation, keyhole, chest

## Abstract

**Background:** Patch-based approaches to regenerating damaged myocardium include epicardial surgical transplantation of heart patches. By the time this therapy is ready for widespread clinical use, it may be important that patches can be delivered via minimally invasive and robotic surgical approaches. This brief research report describes a world-first minimally invasive patch transplantation surgical device design enabled for human operation, master-slave, and fully automated robotic control.

**Method:** Over a 12-month period (2019–20) in our multidisciplinary team we designed a surgical instrument to transplant heart patches to the epicardial surface. The device was designed for use via uni-portal or multi-portal Video-Assisted Thorascopic Surgery (VATS). For preliminary feasibility and sizing, we used a 3D printer to produce parts of a flexible resin model from a computer-aided design (CAD) software platform in preparation for more robust high-resolution metal manufacturing.

**Results:** The instrument was designed as a sheath containing foldable arms, <2 cm in diameter when infolded to fit minimally invasive thoracic ports. The total length was 35 cm. When the arms were projected from the sheath, three moveable mechanical arms at the distal end were designed to hold a patch. Features included: a rotational head allowing for the arms to be angled in real time, a surface with micro-attachment points for patches and a releasing mechanism to release the patch.

**Conclusion:** This brief research report represents a first step on a potential pathway towards minimally invasive robotic epicardial patch transplantation. For full feasibility testing, future proof-of-concept studies, and efficacy trials will be needed.

## Graphical Abstract

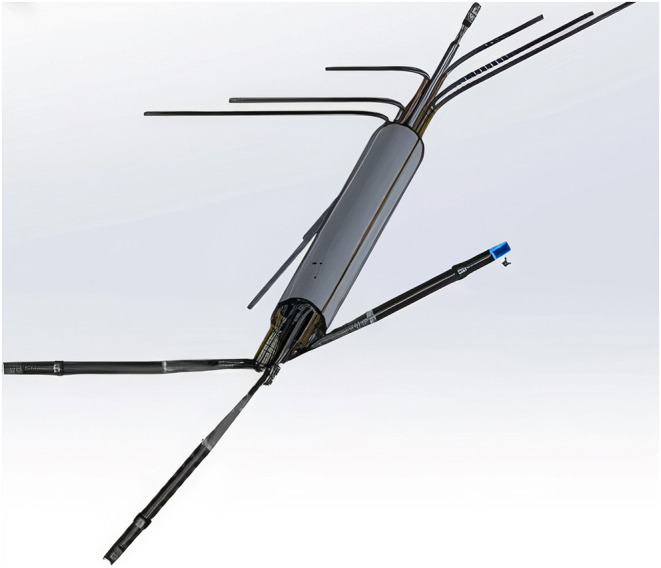

## Introduction

Since the first reports of robotic minimally invasive cardiac surgery (1), there has been increasing attention given to the role of minimally invasive robotics in cardiothoracic surgery (2–6). Meanwhile, tissue engineers have been making gains toward regenerating the myocardium (7–9). The first human trials of patches containing biomaterials/cells applied to the epicardial surface to regenerate the heart have been reported with promising results (10–14). Moreover, increasingly accessible techniques such as 3D bioprinting (one approach to generating heart tissue patches) promise scalability, reproducibility, and highly refined control over the characteristics of the patch to be grafted (7). However, many approaches to regenerate the myocardium surgically using patches applied to the epicardial surface have worked toward a model of open surgery via median sternotomy (8). There is an unanswered but pressing question whether surgical patch-based repair of the heart will need to be delivered by minimally invasive and/or robotic surgery by the time it reaches widespread clinical use (7). Additionally, for heart failure patients who may not be fit for a heart transplant or major surgery but who may tolerate a less invasive keyhole procedure (15), this solution may open up a therapeutic avenue for them.

Our team therefore conceptualised and designed a novel surgical instrument to deliver heart tissue patches to the epicardium. Our multidisciplinary team included a cardiothoracic surgeon, a bioengineer and two specialists in robotics, mechanical engineering, and mechatronics. To our knowledge, the early-stage design we present is a world-first with no similar design existing. This descriptive brief research report represents the initial step on a potentially significant pathway to pivot the field away from its focus on traditional open surgery.

## Method

### Design Process, Objectives, Reasoning

The design process was initiated with several discussions amongst the team to determine the objectives, requirements, and feasibility of the idea. An initial outline of the design was sketched with attention to the ergonomics at human surgery, the size and material requirements for thorascopic insertion and manipulation of the instrument within the chest cavity, the shape requirements to ensure suitability for human cardiothoracic anatomy, the mechanism to allow for an operator to manoeuvre the instrument using handles outside the chest cavity at the proximal (external) end of the instrument and the ability for the device to be controlled in future by both master-slave robotics and fully automated robotics.

Using SOLIDWORKS^®^ (Dassault Systèmes SOLIDWORKS Corp, Waltham, MA, USA) computer aided design (CAD) software, the instrument blueprint was created and revised several times to ensure it was optimised. At this stage, attention was given to the points of attachment for patches onto the instrument and the details of how the patch would fold into the instrument when retracted and then be spread out for deployment when expanded (without damaging the patch). Another challenge was the releasing mechanism for the patch to release it from the instrument. It was decided that tiny attachment nodules/hooks would be placed at the distal ends of the manoeuvrable arms and at the apex of the pyramid created by the three arms converging. When the arms are expanded (outfolded) this stretches out the patch between the metal arms. This has the effect that when outstretched the patch itself would move away from the apex and fold out to form the base of a pyramid.

The patches will be made from alginate 4%/gelatin 8% in cell culture medium, which is a hydrogel that becomes fluid at temperatures over ~28°C and is more solid at lower temperatures. It can be crosslinked ionically by adding calcium chloride (2% w/v in phosphate buffered saline) which increases the strength of the material. A similar hydrogel with a modified molecular structure, gelatin methacryloyl (GelMA), can be used in a similar way to alginate/gelatin but is more robust when it is crosslinked which is done by UV light photocuring. Therefore, we created the instrument design to include areas for attachment nodules/hooks which would be attached to areas within the patch containing small rings of GelMA at the corners and the centre. These reinforced patch ring-corners would be attached to the arms distally. In the folded position, the patch centre will be similarly attached to the instrument platform where the proximal ends of the three arms converge (the apex of the pyramid formed by the arms). When opening the arms (pitch rotational movement) this will pull the patch away from the platform where the proximal ends of the arms converge as it unfolds and expands to become the outstretched base of the pyramid. Next, to release the three patch corners from the apex, the instrument arms can be moved laterally (yaw rotational movement). To ensure that release happens first at the apex/central connection the strength of the GelMA ring will be modified by using fewer layers so that this connection releases first (before the GelMA ring connection to the distal tips of the arms). In case of failure to release by this mechanism, the platforms with the hook/nodule attachments can be moved in a sliding movement distally away from the instrument body, theoretically releasing them by breaking the GelMA rings.

The head has a rotational mechanism (role rotational movement) which allows for the rotation of the patch through 360 degrees. One arm is able to be made shorter than the other two during patch deployment. This means that by opening that arm past 90 degrees to the main instrument body whilst shortening it, the patch should be able to open and face any lateral direction (similar to the triangle that can be made with the extended index and middle finger to the thumb in opposition). This gives the instrument three degrees of rotational movement in addition to the three degrees of translational movement in the X, Y, and Z axis which are achieved by movements at the entry into the chest (similar to moving a pencil pinched lightly between the thumb and index finger). Additional to these six degrees of freedom, each arm is capable of pitch and yaw rotation individually. The shortenable arm has the additional benefit of being openable in a confined space, for example, if facing the surface of the heart when opening, so that its excursionary movement can be completed without damaging surrounding structures. Overall, these movements will allow for the patch to be expanded in the hemithorax and then rotated to face the surface of the heart at the correct angle.

One surgical approach for the operation of this instrument is via standard left-sided anterolateral multiportal video-assisted thorascopic surgery (VATS)—similar to a left lower lobectomy approach but with the left lung deflated via endobronchial intubation and single lung ventilation of the right lung. The pericardium would need to be partly opened to gain access to the epicardial surface. In particular, for a chronic ischaemic cardiomyopathy heart failure patient or after myocardial infarction (MI), the target area may be the anterolateral surface of the heart over the left ventricle. With the rotational head and the releasing mechanism, it should be possible to manoeuvre the patch and apply it to the epicardium on most surfaces reachable without moving the heart: a minimally invasive VATS transplantation of a regenerative cardiac patch.

Following these discussions and revisions aimed at optimising the instrument design we 3D printed a version of the instrument to assess for size and identify learning points. This “sizing and learning” print was in preparation for the full metal prototyping which will use 17-4 Ph Stainless Steel (SAE Type 630 stainless steel—hardened stainless steel containing ~15% chromium, 5% nickel, 5% copper).

## Results

### Early-Stage Design Outcomes

**Figure 1:**
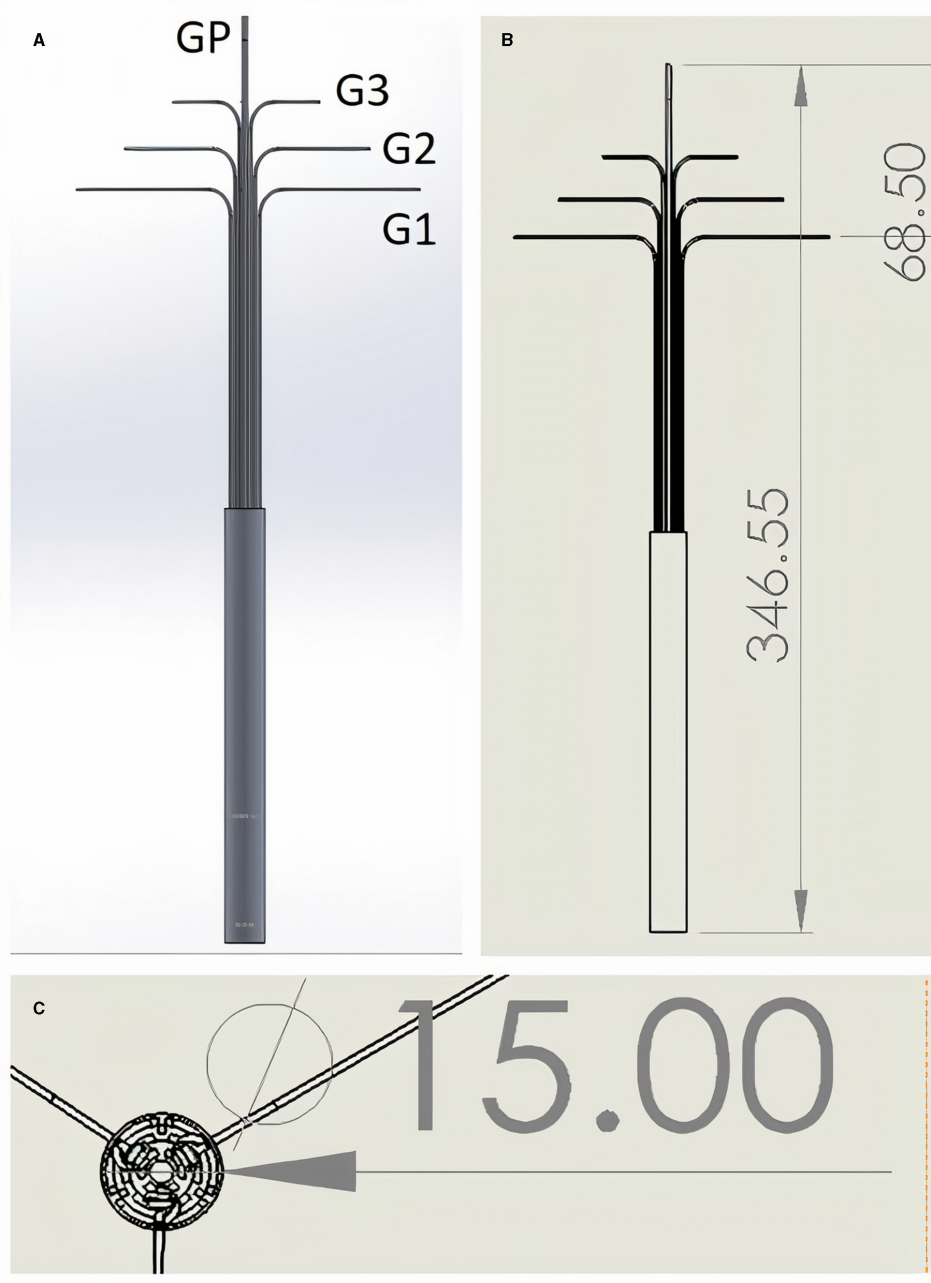
**(A,B)** Lateral view. One single Grip (GP) controls forward protrusion and backwards retraction of the instrument. Grips G1–G3 are triplicate (only two of each are visible in the 2D lateral view images shown), and control each of the three distal arms to which they are attached (distal arms not visible - infolded and covered by sheath). G3 Grips control the curvature of the patch, G2 the rotation angle of the arm, and G1 the deployment angle of the arm. **(C)** “Top down” view showing triplicate arrangement of Grips when in line with each other and opened with 120 degree angles between each set of three Grips. Grips exit the cylindrical instrument body which has diameter of 15 mm. All measurements in mm. See Supplementary Video 1 for dynamic demonstration *in silico*.

The device has nine Grips plus one push-out Grip (Figure 1, Supplementary Video 1). Each arm has three Grips (Figure 1A, Labels G1, G2, and G3). The top Grip (G3) controls the attachment platforms and therefore the curvature of the patch (it also acts as a releasing mechanism if attachment platforms are moved beyond the maximal boundary of the outfolded patch), the middle Grip (G2) controls the rotation angle of the arm, and the bottom Grip (G1) controls the deployment angle of the arm. The push out Grip (GP) protracts or retracts the arms from their sheath. Supplementary Figure 1 shows a frontal and trimetric view of the mechanism with the sheath removed.

The designed length of the instrument was 35 cm and cylinder diameter was 1.5 cm (Figures 1B,C). Each arm was 60 mm, thus the maximum size of a triangular outfolded patch would be ~18 cm^2^. The tips of each arm are 11.2 cm apart while opened at 90 degrees to the body of the instrument. The smallest parts in our instrument were the joints which are cylindrical type joins (which act like screws connecting two linked pieces) and these were 1 mm diameter and 1 mm height.

**Figure 2:**
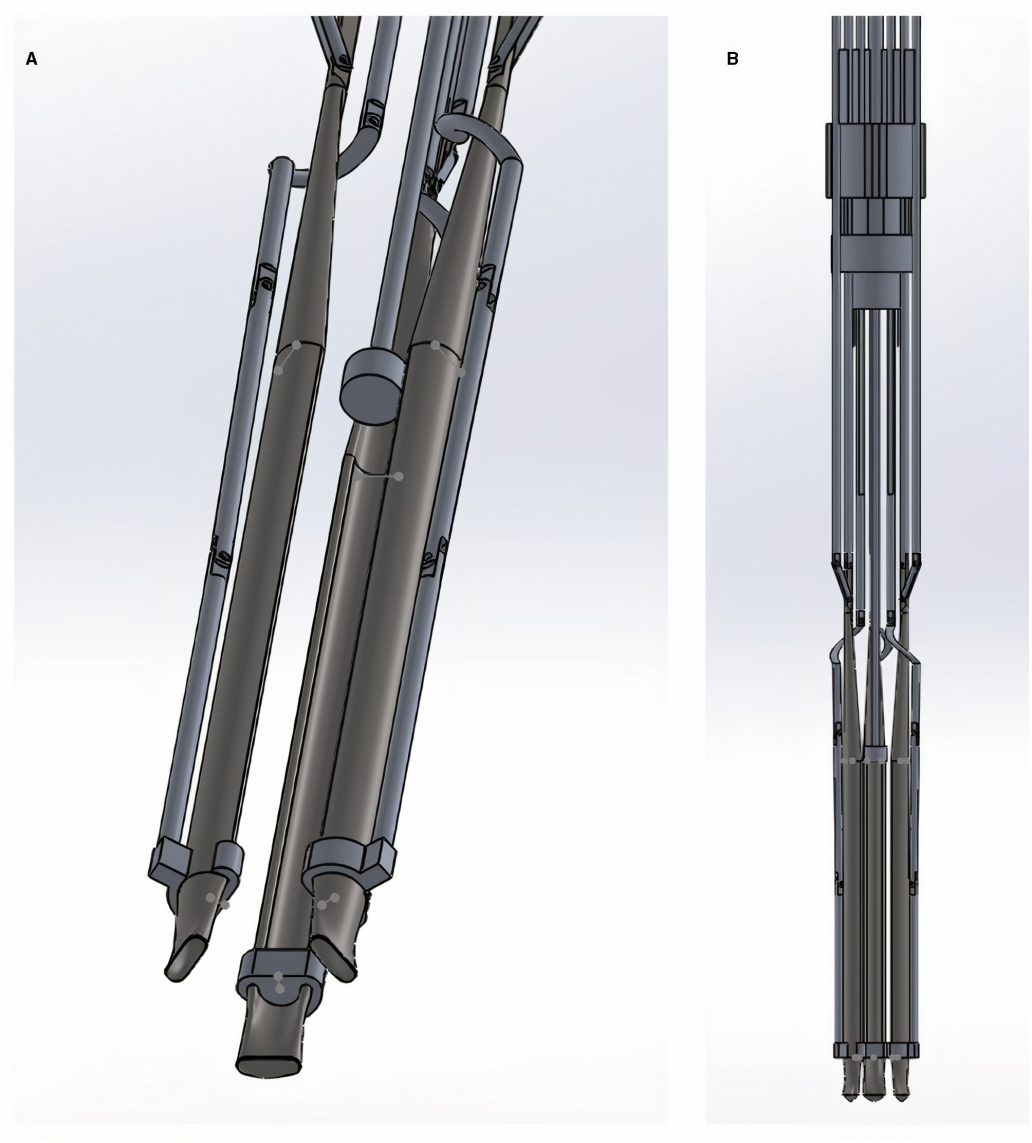
**(A,B)** Close-up angled view **(A)** and complete lateral view **(B)** of the distal end of the instrument (the patient end where the patch would be enclosed). Movements of this compartment are controlled at the other (operator's) end of the instrument by the connected Grips shown in Figure 1.

Special features of the instrument included a space between the arms when infolded where the folded patch could be stored prior to deployment (Figure 2). The patch could therefore be inserted into the chest within the mechanism (and covered by the outer sheath) without damaging it during insertion. Control over each of the three arms was achieved by three separate mechanisms connecting the arms to the Grips at the proximal end of the device (Supplementary Figure 2).

The arms were designed so that they could be individually rotated, including when the arms are folded out from their closed position. The region conveying rotationary control is shown in Supplementary Figure 3. In Supplementary Figure 3B the proximal (operator's end) region where the Grips converge is shown.

**Figure 3:**
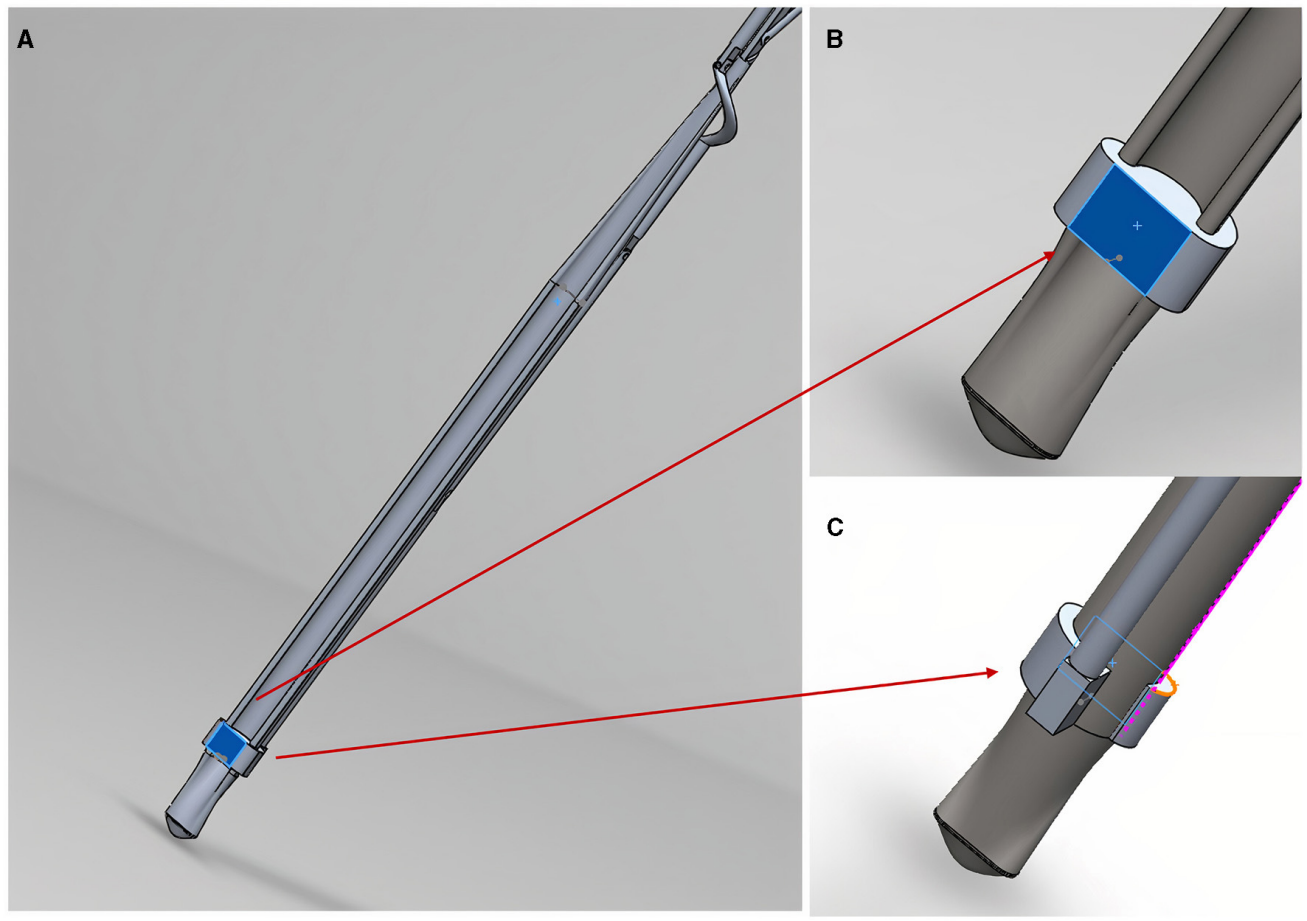
The patch releasing mechanism in close-up. **(A)** Shows an arm patch attachment point/platform (highlighted in blue); **(B)** shows anterior view close-up of the platform where a hook/nodule (not shown) will attach patch corners; **(C)** shows a posterior view of the platform shown in **(B)**.

Along each arm the design includes a moveable attachment platform which can be controlled using Grip number 3 (control pathway highlighted in Supplementary Figure 2A). Supplementary Video 1 shows the releasing mechanism as it moves along one of the three arms. This movement from the distal aspect of an arm to the proximal aspect allows for the attachment platforms to be moved, pulling them away from the patch connections and releasing the patch. These patch connecting platforms are shown in more detail in Figure 3.

**Figure 4:**
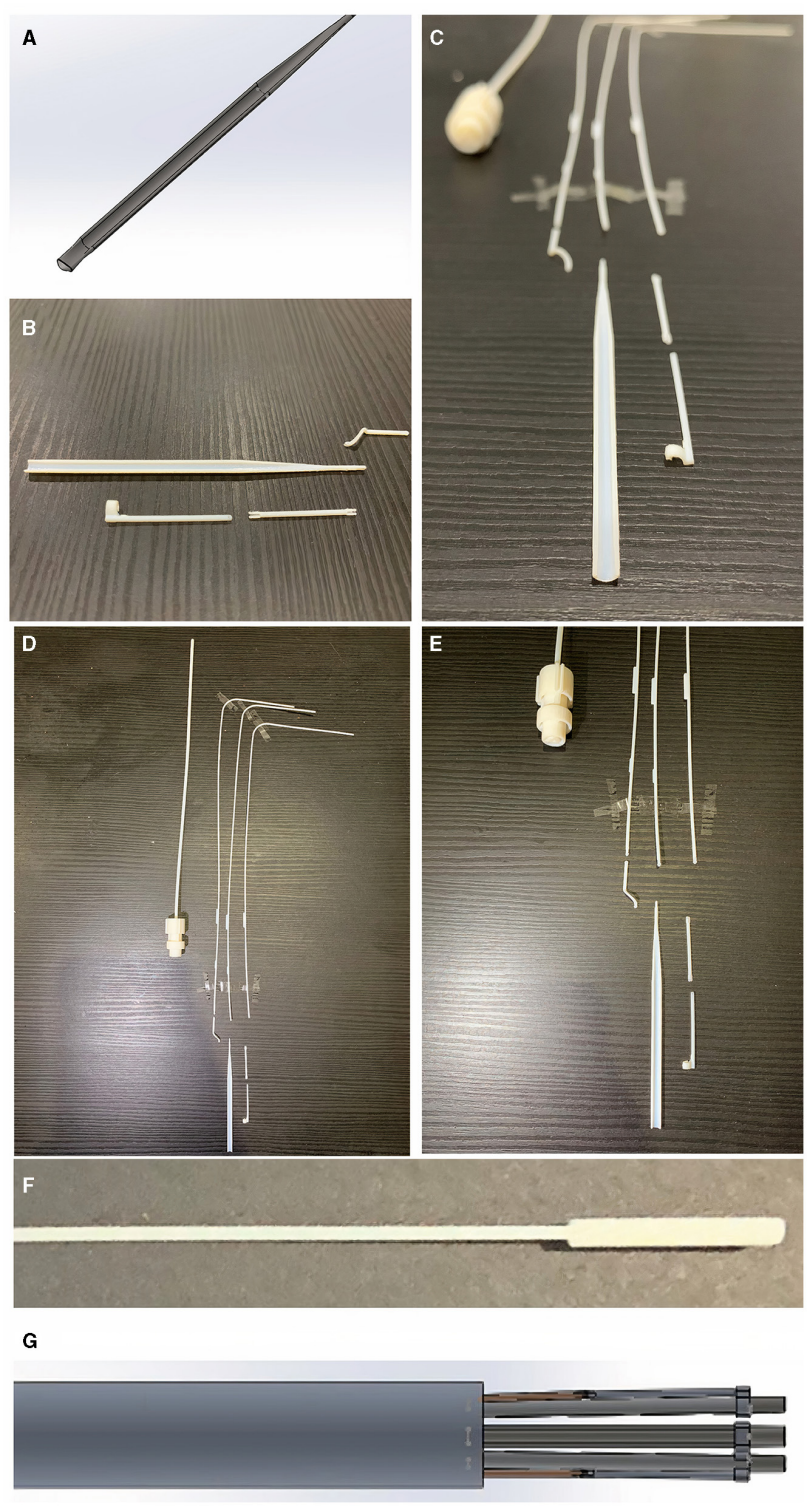
Curved edges of the arm to reduce risk of injury to surrounding structures inside the chest. **(A)** Shows the CAD blueprint for the main component of one of the arms. The photographs in **(B,C)** show the same curved edged arm with surrounding component parts after the sizing and learning resin print (VeroVivid and Agilus). These parts were reproduced with a high degree of accuracy to the blueprint and the surrounding component parts fitted with the main body of the arm. This suggests these components will be accurately fabricated in the subsequent stainless steel prototype. Photographs in **(D,E)** show component parts outcome of Agilus/VeroVivid resin sizing and learning print. Despite inherent printer error margin, sizes were accurate to the CAD blueprint and appropriate for surgical use. There were limitations to the resolution printable by this method shown by the fusion of resin material at the distal tip in the photograph **(F)** compared to the input CAD image shown in **(G)**. This has shown that subsequent prototyping will require a manufacturing method capable of retaining the detail of small parts within the device.

The attachment platforms will have small hooks (not shown in the figure) where they will be able to attach to rings of a semi-robust crosslinked hydrogel (GelMA) at the corners of the patch. If the releasing mechanism fails to move the hook from the patch and release that corner, the arm could be rotated using the rotationary mechanism shown in Supplementary Figure 3 to pull the connection away from the patch. To reduce the risk of injury to surrounding structures, the edges of the design are curved and smooth (Figure 4).

### 3D Printing of Sizing and Learning Resin Prototype

Some of the parameters for the 3D printer settings for the sizing and learning prototype are shown in Supplementary Figure 4. We used a Stratasys J750™ polyjet multi-material 3D printer (Stratasys, Eden Prairie, MN, USA). The materials used to print the test (sizing and learning) prototype parts were VeroVivid (a translucent colour material) and Agilus (an elastomeric polymer) which cost under £10 GBP ($14 USD). The total cost (excluding hardware purchase) of the sizing and learning print was < £60 GBP ($83 USD). A comparison of the resin size to the computer-aided design blueprint is shown in Figure 4. The printed product underwent a chemical bath (in a solvent named Opteon SF-79, which is used to dissolve the support material for the printed parts) and during this final phase some of the tiny cylinder joints (6 out of 13) were lost.

## Discussion

### Brief Research Report key Considerations and Unanswered Questions

Our novel surgical instrument design is aimed at minimally invasive approaches to transplant patches for myocardial regeneration and is enabled for future robotic control of the device. Whilst it has not been designed to fit with current commercially available cardiothoracic surgical robots, it has been designed to be ready for robotic control, where the instrument itself would be attached as a forearm to a robotic arm. This was based on the capabilities in our department to build a full robotic arm and the instrument could be used for master-slave or full automation. It is important that any new instrument design is enabled for compatibility to these envisaged future robotic controls.

The sizing and learning resin 3D print gave several insights which will be invaluable for the full prototyping phase from stainless steel. Firstly, it showed us that a major challenge is going to be accounting for the manufacturing machine error with such small parts (our smallest components are 1 mm diameter × 1 mm height cylinder joints). The printer we used has a high resolution (horizontal build layers down to 14 μm) but there is also print error margin of ±150–200 μm (up to 1% of the diameter of our instrument and 20% of our 1 mm cylinder joints). These valuable learning points taught us that the next phase will likely require both a slight enlarging of the instrument and also the use of a very low error manufacturing technique for the stainless steel prototype. Furthermore, each part in the design is a perfect fit and therefore allowed no space for collision volume (the distance away from the molecular centre which may come into contact with adjacent parts). The metal print in future will require micro-adjustments across every part of the design to add ±100 μm space around each part so that there is room for manufacturing machine error in the generation of the stainless steel prototype.

Another consideration is that following the initial fabrication of the instrument it will have to be immersed in the same chemical bath used for the resin sizing and learning print (Opteon SF-79) during which time our tiny joints can still be lost. In fact, during the resin learning print 6 of our 13 joints were lost during the chemical bath. Therefore, we may need to include over 20, adding in extra joints in anticipation that some will be lost during the manufacturing process. Future trials by our group will determine whether these issues can be minimised by increasing the size of the instrument without removing the clinical utility.

We have envisaged the instrument being used for VATS approaches and it should be highly versatile so it can be used with multiple surgical approaches via uniportal (one large “keyhole” in the chest for all instruments) or multiportal (several keyhole incisions) VATS—for example with a left anterolateral approach. The exact approach would depend on the target area of the heart (for instance we would probably want to deploy the patch over a specific infarcted or failing area of the left ventricle). This will likely need a wide space and multiple ports to get a good view and manoeuvre into the best position.

The patch itself (see Supplementary Video 1) can be customised in many ways with different biomaterials to control the viscosity of the patch and also for different cell types within the patch (16). Many different cells have been tried by researchers in this field, often derived from stem cells (7). Our approach might be able to treat heart failure for patients who would otherwise not be eligible for a transplant (in a less invasive standalone procedure to patch the myocardium rather than replace the whole heart). There are many complex considerations for this, including whether one could generate a patch of patient-specific heart tissue from stem cells reprogrammed from the patient's own skin cells and transplant that (7). Importantly, all approaches to myocardial regeneration with a patch have so far have imagined an open surgical transplantation method which may actually preclude this treatment in many of the heart failure patients it is ultimately intended to benefit.

The therapeutic approach will be different for the acute vs. chronic phase of ischaemic cardiomyopathy and/or MI and initially this instrument has been designed with a view to being applicable in a non-acute situation as a standalone procedure. In the acute phase it may not even be required to transplant cells but just putting a patch as an adjunct to regular treatment which stimulates macrophages and other inflammatory responses may be beneficial for remodelling and cardiac function after MI (17). For this instrument, it is clinically most likely to be useful for chronic heart failure caused by ischaemia or MI. There are many open questions and a large amount of research is focused on regenerating the myocardium (7, 8). The unique selling point of this instrument is that no one has yet presented a solution to the question of how to transplant patches without open surgery. By the time regenerative patches for the myocardium are ready for clinical use they may need to be able to be transplanted by minimally invasive and/or robotic approaches. If patch transplant were to be used as an adjunct in a patient already undergoing another procedure, a minimally invasive method would need to exist because the primary procedure may not be via open surgery. As a standalone treatment for high-risk patients with heart failure who cannot have open surgery, it may be beneficial for them if this can be done by a less invasive approach.

This brief research report represents the first time this approach has been presented (without restriction for anyone to build upon). Whilst it has been developed for cardiac applications, it could even be co-opted for other applications (e.g. abdominopelvic) where diverse minimally invasive robotic approaches are also being developed (5, 6). Its main limitation is that the descriptive work herein is at an early stage. It is likely that future instrument designs will have to be larger, perhaps more fitting for a 5–6 cm incision rather than a 2 cm one. This would also have the potential benefit of bypassing the major challenge of how to infold a patch then outfold it like a net (without crushing it). Specifically, a 5–6 cm diameter instrument could potentially accommodate a non-folded patch large enough to be used without repeated application of small patches. Future studies will be needed to optimise designs, fully prototype them and then assess the actual performance of any prototype in proof-of-concept surgery followed by full *ex-vivo* (cadaver) and *in-vivo* trials. Efficacy will need to be evaluated in terms of transplantation success over repeated applications (with full measurement of parameters such as time to delivery, deployment accuracy in a non-beating and beating heart, patch size, covered epicardial surface area and a full range of quantitative and qualitative analyses—all compared to relevant controls). Then trials will be needed for a functional demonstration of a clinically and statistically significant improvement in cardiac function (including non-inferiority against the alternative approach of traditional open surgery). Whilst this brief report article has focused on a surgical instrument design, significant work will also be needed to show that the patch matrix we propose—alginate 4%/gelatin 8% patches ± cells based on our previous optimisation for cardiac applications (9, 16, 18)—is superior to reference patch materials. Translating these technologies is a lengthy process, which is part of the point: it should happen in parallel to the advancements currently underway in the field of myocardial regeneration, or the field risks unveiling a successful new treatment to a world that might have moved away from traditional open surgery (2, 19), limiting how to actually deliver it.

## Conclusion

Over 12 months our multidisciplinary team has invented a design for a novel surgical instrument which is at the leading edge of innovation in this field. Findings from our sizing and learning resin print of this instrument have prepared the way for the stainless-steel prototype to be manufactured. This is a world-first achievement which may alter the direction of research for surgical transplantation of patches for myocardial regeneration. This brief research report presents the first step on this pathway, for which further trials will be needed.

## Data Availability Statement

The original contributions presented in the study are included in the article/Supplementary Material, further inquiries can be directed to the corresponding author/s.

## Author Contributions

YZ: conceptualization, data curation, formal analysis, investigation, methodology, resources, software, validation, visualization, and writing—review and editing. CR: conceptualisation, data curation, formal analysis, funding acquisition, investigation, methodology, project administration, supervision, validation, writing—original draught, and writing—review and editing. CG and LZ: conceptualisation, data curation, funding acquisition, methodology, project administration, supervision, and writing—review and editing. All authors contributed to the article and approved the submitted version.

## Funding

CR was supported by a Sir John Loewenthal Scholarship 2019 (University of Sydney), the Le Gros Legacy Fund New Zealand [PhD012019] and a Heart Research Australia Scholarship [PhD2019-02]. CG was supported by a University of Sydney Kick-Start Grant, University of Sydney Chancellor's Doctoral Incentive Programme Grant, a Sydney Medical School Foundation Cardiothoracic Surgery Research Grant, UTS Seed Funding and the Catholic Archdiocese of Sydney Grant for Adult Stem Cell Research (2019).

## Conflict of Interest

The authors declare that the research was conducted in the absence of any commercial or financial relationships that could be construed as a potential conflict of interest.

## Publisher's Note

All claims expressed in this article are solely those of the authors and do not necessarily represent those of their affiliated organizations, or those of the publisher, the editors and the reviewers. Any product that may be evaluated in this article, or claim that may be made by its manufacturer, is not guaranteed or endorsed by the publisher.

## Supplementary Material

Supplementary Figure 1 The instrument with the sheath covering the distal arms removed. Frontal **(A)** and trimetric **(B)** views show the instrument with arms in the infolded position.

Supplementary Figure 2 Control pathways linking the operator's end to the distal arms. **(A–C)** Highlighted views of the three Grips controlling the distal arms.

Supplementary Figure 3 Mechanism allowing for adjustment of the rotational angle of the arms. Infolded arms **(A)** can be outfolded to open position and rotated individually by the grips shown in **(B)**. View shown in **(A)** is the distal (patient's end) and **(B)** shows a “top down” view of the proximal (operator's end) of the instrument (looking down onto the Grips).

Supplementary Figure 4 3D printing parameters for the resin sizing and learning prototyping of individual parts.

Supplementary Video 1 Video walkthrough of patch delivery device, including background and mechanistic demonstration from the computer-aided design (CAD).
