# Supplementary material for: A World-First Surgical Instrument for Minimally Invasive Robotically-Enabled Transplantation of Heart Patches for Myocardial Regeneration: A Brief Research Report
Source: Front Surg. 2021 Oct 6;8:653328. doi: 10.3389/fsurg.2021.653328 (PMC8526867; doi:10.3389/fsurg.2021.653328)
Supplement: Supplementary file 2 [file Data_Sheet_2.pdf]

Project Members

Chris Roche, full time USYD PhD student and cardiac surgical trainee (clinical work paused to do PhD). Official appointment at UTS is as a 'visiting scholar'; primary PhD candidature is with the University of Sydney as a PhD student supervised by Carmine Gentile.

Yiran Zhou, final year UTS mechanical engineering student, BEng(Mech)(Hons) – Capstone project UTS student supervised by Liang Zhao.

Carmine Gentile, Lecturer, School of biomedical engineering, Faculty of Engineering and IT, UTS. Retains his supervisor role to Chris Roche via an honorary USYD Lecturer role but has primary lecturer role at UTS.

Liang Zhao, Senior Lecturer, School of Mechanical and Mechatronic Engineering, Faculty of Engineering and IT, UTS. Supervisor of Yiran Zhou.

Contributions and Individual Roles to Date

Chris Roche – Conceptualisation of the original idea. Development of the instrument design, in particular contributing bioengineering knowledge (as the device is designed to deploy patches similar to those generated as the primary focus of his PhD). Also contributed cardiac surgery clinical knowledge including the original idea of trying to find a way to deliver patches by a minimally invasive cardiac surgical instrument as well as aspects of the instrument required for it to be used clinically at surgery and for it to be able to deploy 3D bioprinted patches of heart tissue by minimally invasive cardiothoracic surgical approaches. He did the work of writing up the (unpublished) scientific report/paper first draft and created the associated video attached to this work.

Yiran Zhou – development of the design, in particular he contributed knowledge about how the mechanism of the instrument could work. He also did the work of turning the design comments/ideas/objectives from the team during the design process into the SolidWorks Computer Aided Design blueprint for the instrument and he also did the work of the prototyping/3D printing of the resin prototype (and will also do the work of turning the SolidWorks CAD data into a stainless steel prototype for the next phase of initial prototyping). He contributed all the data used to generate the figures of the scientific report/paper first draft (SolidWorks illustrations and moving video demonstrations, printer settings and photographs of the outcome of the first resin 3D print of the first prototype). He contributed significantly to the first draft of the manuscript by providing the biomechanical engineering details and parameters which Chris Roche did not have knowledge of.

Carmine Gentile – development of the design, in particular contributing bioengineering/biomaterials knowledge about the patch the instrument is designed to deploy, for example about how the patch could be better designed to fit the instrument releasing mechanism. He reviewed the manuscript written by Chris Roche and Yiran Zhou which is associated with project. He provides supervision to Chris Roche as his PhD supervisor with the primary focus of that PhD being the 3D bioprinted patches themselves.

Liang Zhao – development of the design, in particular contributing knowledge about which biomechanical approaches could work, how to refine the instrument so that the design is optimised including overcoming challenges to do with adding in additional degrees of movement and control. Also contributed robotics and automation knowledge, for example how to ensure instrument is robotically-enabled for potential future attachment to full robotic control hardware as well as guidance to Yiran Zhou on generating the prototype. He reviewed the manuscript written by Chris Roche and Yiran Zhou which is associated with this project. He provided supervision to Yiran Zhou as his Capstone Project supervisor.
