# Supplementary figures and images for: A World-First Surgical Instrument for Minimally Invasive Robotically-Enabled Transplantation of Heart Patches for Myocardial Regeneration: A Brief Research Report
Source: Front Surg. 2021 Oct 6;8:653328. doi: 10.3389/fsurg.2021.653328 (PMC8526867; doi:10.3389/fsurg.2021.653328)

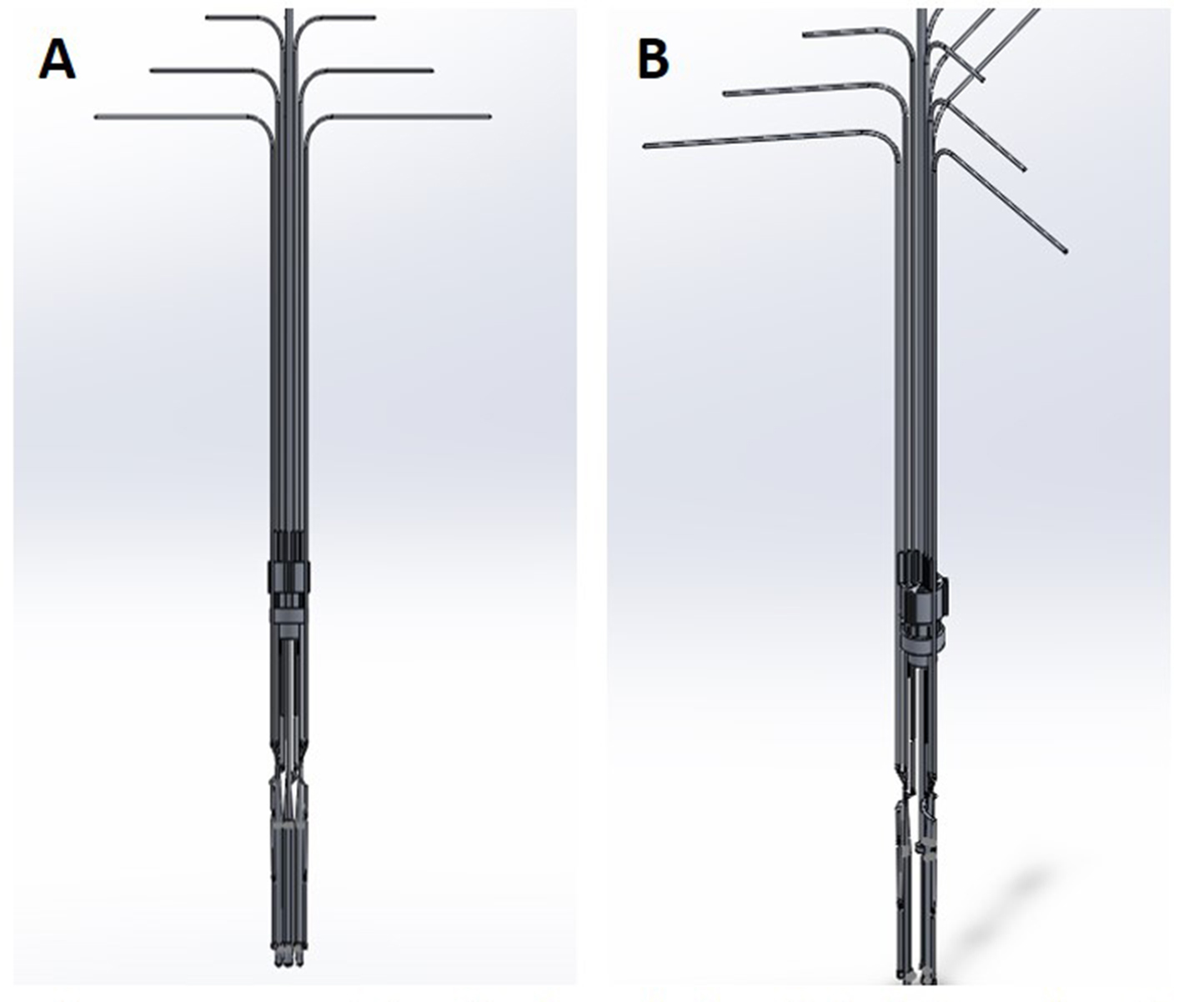

Supplement: Supplementary Figure 1 — The instrument with the sheath covering the distal arms removed. Frontal (A) and trimetric (B) views show the instrument with arms in the infolded position. [file Image_1.jpeg]

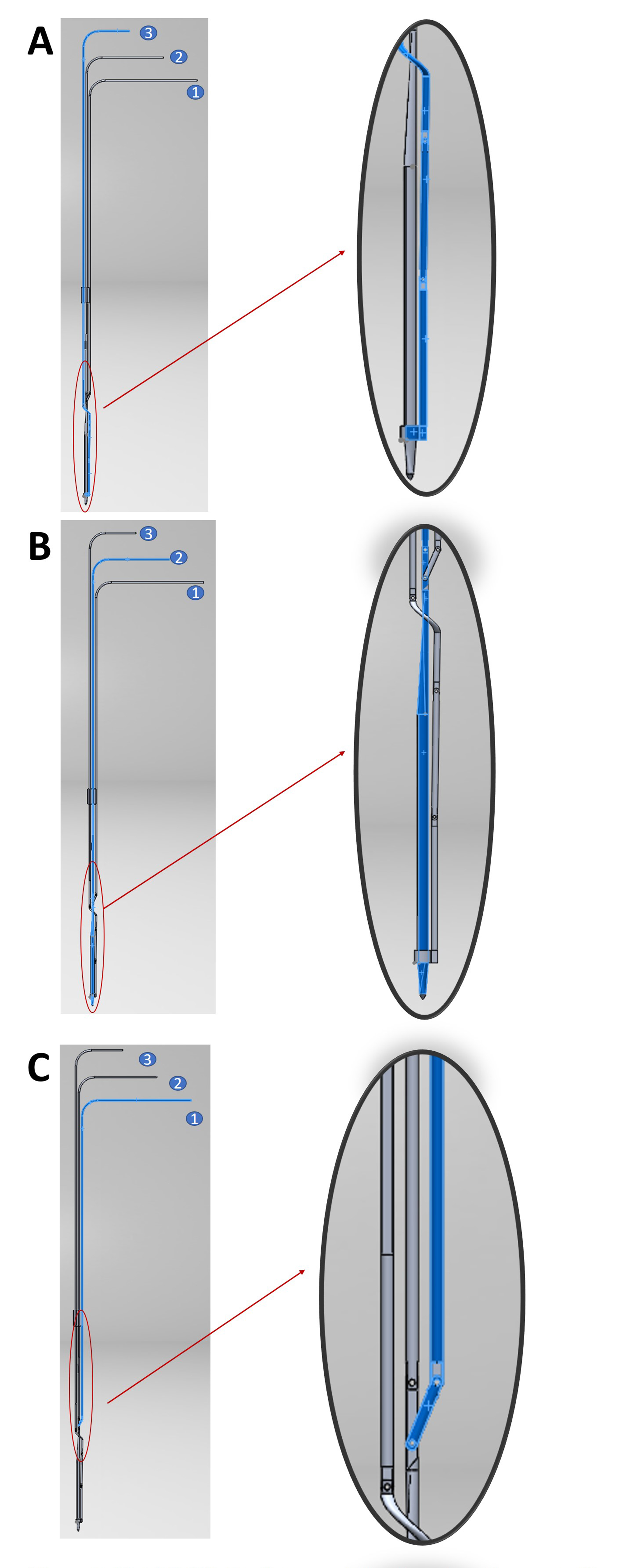

Supplement: Supplementary Figure 2 — Control pathways linking the operator's end to the distal arms. (A–C) Highlighted views of the three Grips controlling the distal arms. [file Image_2.jpeg]

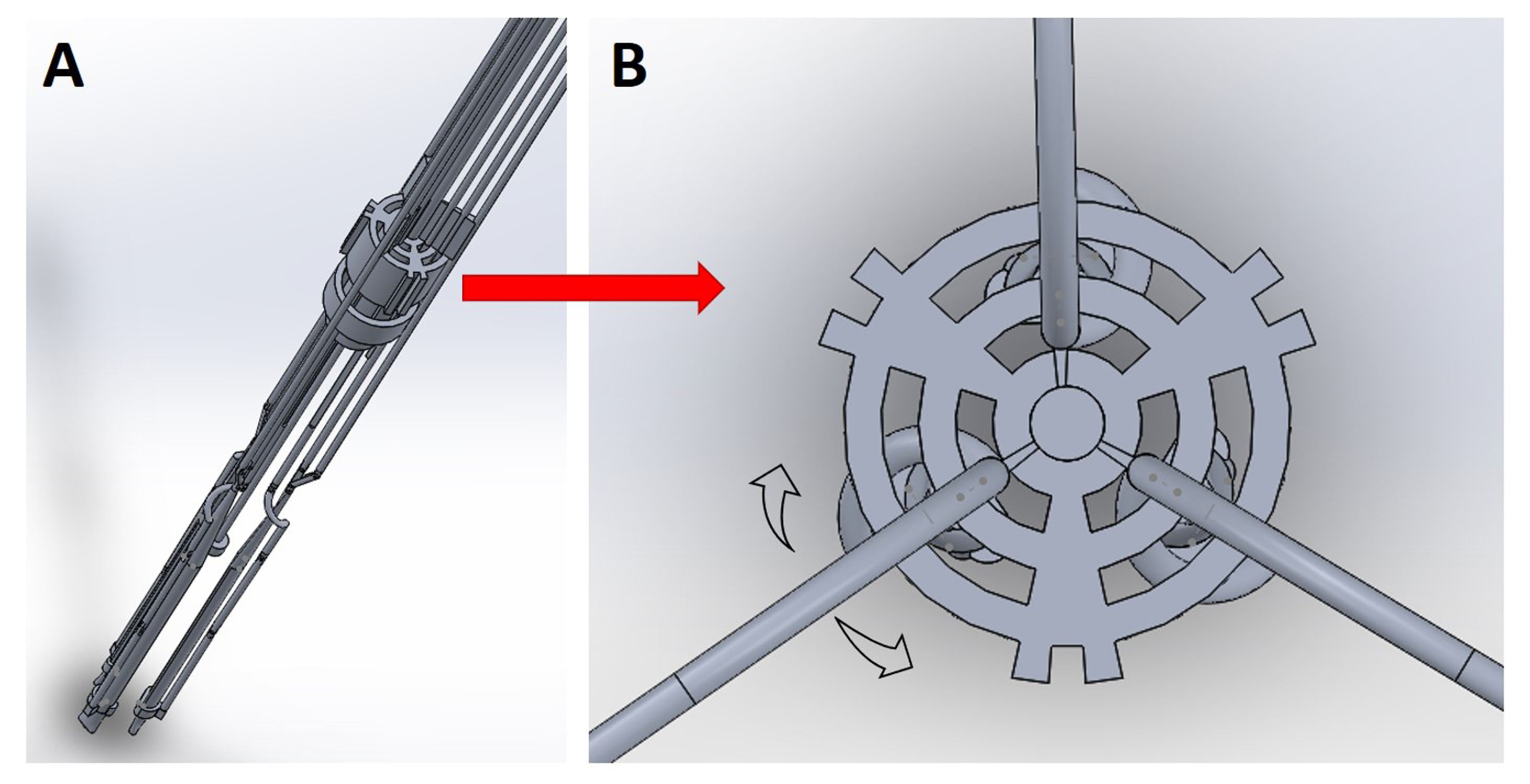

Supplement: Supplementary Figure 3 — Mechanism allowing for adjustment of the rotational angle of the arms. Infolded arms (A) can be outfolded to open position and rotated individually by the grips shown in (B). View shown in (A) is the distal (patient's end) and (B) shows a “top down” view of the proximal (operator's end) of the instrument (looking down onto the Grips). [file Image_3.jpg]

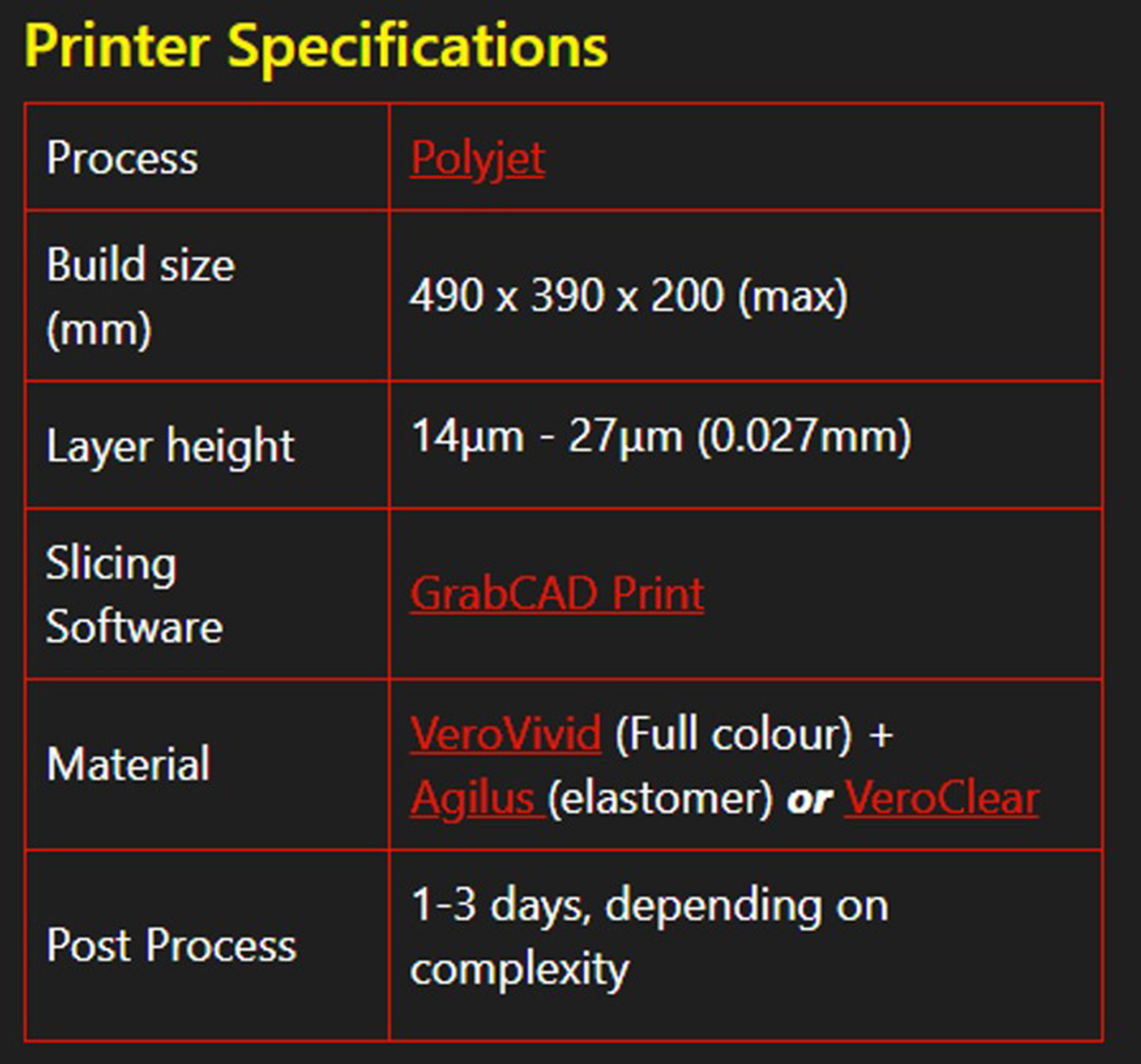

Supplement: Supplementary Figure 4 — 3D printing parameters for the resin sizing and learning prototyping of individual parts. [file Image_4.jpg]
